# Low muscle strength and low phase angle predicts greater risk to mortality than severity scales (APACHE, SOFA, and CURB-65) in adults hospitalized for SARS-CoV-2 pneumonia

**DOI:** 10.3389/fnut.2022.965356

**Published:** 2022-12-22

**Authors:** Oscar Rosas-Carrasco, Gisela Núñez-Fritsche, Miriam Teresa López-Teros, Pamela Acosta-Méndez, Juan Carlos Cruz-Oñate, Ada Yuseli Navarrete-Cendejas, Gerardo Delgado-Moreno

**Affiliations:** ^1^Department of Health, Universidad Iberoamericana, Mexico City, Mexico; ^2^General Hospital Penjamo, Guanajuato, Mexico

**Keywords:** phase angle, sepsis-related organ failure assessment (SOFA), score for pneumonia severity (CURB-65), APACHE II, dynapenia, low grip strength, sarcopenia

## Abstract

**Introduction:**

The acute physiology and chronic health evaluation (APACHE), sepsis-related organ failure assessment (SOFA), score for pneumonia severity (CURB-65) scales, a low phase angle (PA) and low muscle strength (MS) have demonstrated their prognostic risk for mortality in hospitalized adults. However, no study has compared the prognostic risk between these scales and changes in body composition in a single study in adults with SARS-CoV-2 pneumonia. The great inflammation and complications that this disease presents promotes immobility and altered nutritional status, therefore a low PA and low MS could have a higher prognostic risk for mortality than the scales. The aim of the present study was to evaluate the prognostic risk for mortality of PA, MS, APACHE, SOFA, and CURB-65 in adults hospitalized with SARS-CoV-2 pneumonia.

**Methodology:**

This was a longitudinal study that included *n* = 104 SARS-CoV-2-positive adults hospitalized at General Hospital Penjamo, Guanajuato, Mexico, the PA was assessed using bioelectrical impedance and MS was measured with manual dynamometry. The following disease severity scales were applied as well: CURB-65, APACHE, and SOFA. Other variables analyzed were: sex, age, CO-RADS index, fat mass index, body mass index (BMI), and appendicular muscle mass index. A descriptive analysis of the study variables and a comparison between the group that did not survive and survived were performed, as well as a Cox regression to assess the predictive risk to mortality.

**Results:**

Mean age was 62.79 ± 15.02 years (31–96). Comparative results showed a mean PA of 5.43 ± 1.53 in the group that survived vs. 4.81 ± 1.72 in the group that died, *p* = 0.030. The mean MS was 16.61 ± 10.39 kg vs. 9.33 ± 9.82 in the group that died, *p* = 0.001. The cut-off points for low PA was determined at 3.66° and ≤ 5.0 kg/force for low grip strength. In the Cox multiple regression, a low PA [heart rate (HR) = 2.571 0.726, 95% CI = 1.217–5.430] and a low MS (HR = 4.519, 95% CI = 1.992–10.252) were associated with mortality.

**Conclusion:**

Phase angle and MS were higher risk predictors of mortality than APACHE, SOFA, and CURB-65 in patients hospitalized for COVID-19. It is important to include the assessment of these indicators in patients positive for SARS-CoV-2 and to be able to implement interventions to improve them.

## Introduction

In December 2019 in the city of Wuhan China, an outbreak of pneumonia was recorded, caused by the SARS-CoV-2 virus, also known as Coronavirus-19, associated with high morbidity and mortality (1). The SARS-CoV-2 infection produces an inflammatory process characterized by an increase in inflammatory cytokines such as tumor necrosis factor-alpha (TNF-α), interleukins 1 and 6 (lL-1 and IL-6), and C-reactive protein (CRP), among others. This inflammation, together with the immobility and inadequate nutrition seen in patients hospitalized for pneumonia, contributes to the loss of muscle mass and strength (2, 3).

Due to the high mortality rates resulting from SARS-CoV-2 infection in people with risk factors and unvaccinated, early markers are needed to help the clinical team identify patients at high risk of death in order to implement timely interventions to reduce this risk. So far, different disease severity and mortality risk scales have been applied in hospitalized critically ill patients: the acute physiology and chronic health evaluation II (APACHE II), the sepsis-related organ failure assessment (SOFA), and the score for pneumonia severity (CURB-65) (4, 5). These scales have proven useful for predicting mortality in SARS-CoV-2-infected patients (6).

On the other hand, other predictors of mortality in hospitalized patients with SARS-CoV-2 infection have been proposed, such as the phase angle (PA), obtained from bioelectrical impedance analysis (BIA) (7–9). PA is an effective marker to detect health conditions and take preventive action, as well as a predictor of mortality, morbidity, length of hospital in patients with COVID-19 (9–11). Muscle strength (MS) is another predictive marker to mortality and longer hospital stay in patients hospitalized for COVID-19 (11, 12). These recently published studies show the importance of continuing to study markers with better predictive capacity to mortality in hospitalized patients with SARS-CoV-2. At present, these severity scales as low PA and MS, have been shown to be predictive of mortality, but individually, no study has shown which represents a greater risk of mortality in the same group of patients. The great inflammation, support ventilation, use the tubes and catheters and others; promotes immobility, loss of appetite, increased caloric expenditure and others factors together impact nutritional status (including the body composition and MS), therefore a low PA and low MS could have a higher prognostic risk for mortality than the severity scales.

Therefore, the aim of this study is to determine if low MS and PA predicts greater risk to mortality than severity scales (APACHE, SOFA, and CURB-65) in a group of adults hospitalized for SARS-CoV-2 pneumonia.

## Materials and methods

### Study design and population

This is a longitudinal, prognostic, cohort study that included 104 SARS-CoV-2-positive adults hospitalized at General Hospital Penjamo, in the state of Guanajuato, Mexico during the COVID-19 pandemic, from August to December, 2020. All patients admitted with a diagnosis of SARS-CoV-2-positive pneumonia of any degree of severity were included. The diagnosis of pneumonia was made by the medical team in charge of the hospitalization and supported by plain computed axial tomography (CAT) and/or chest tele radiography. Participants with pacemaker or defibrillator, pleural, peritoneal, legs oedema, renal replacement therapy (hemodialysis or peritoneal dialysis, current intake of diuretics, presence of fever, and diarrhea were excluded from the study, resulting from the use of BIA).

### Measurements

Mortality: the number of deaths that occurred during the follow-up period was recorded.

Phase angle: it was assessed using the BIA Quantum V device, by RJL Systems. The crude data were obtained for resistance (R), reactance (Xc), and PA at a frequency of 50 Hz. All the participants were evaluated within the first 24 h of admission, lying down, with the bed tilted at 45°, electrodes were placed on the both sides of the body on the dorsal surface of the metacarpophalangeal and metatarsophalangeal joints, medially between the distal prominences of the radius and ulna and between the medial and lateral malleoli at the ankle. The cut-off point for low PA (≤ 3.66°), considering the fifth percentile (−SD 1.65) as the lower limit of normality based on previous studies and considering that this study included adults aged from 31 to 96 years, men and women hospitalized with pneumonia due to COVID-19 (9).

Muscle strength: it was evaluated by the Takei 5001 Hand Grip Analog Dynamometer, all participants were evaluated within the first 24 h of admission, lying down with your bed tilted at 90°; with the elbow flexed at 90°, the test was carried out three times from each arm and the highest score was considered for the analysis. The cut-off point for low grip strength ≤ 5.0 kg/force was determined by the 20th percentile of the total data of the sample included in this study and considering that this study included adults aged from 31 to 96 years, men and women hospitalized with pneumonia due to COVID-19 (13).

Body composition and anthropometric variables: fat mass index (FMI) and appendicular muscle mass index (AMMI) were assessed using the aforementioned BIA (Quantum V, RJL Systems, MI, USA), specific equations were used in the study population to assess FMI and AMMI (14). Body weight (kg), height (meters) and body mass index (BMI), i.e., weight/height^2^, were also assessed.

Disease severity scales: CURB-65 (5) is a severity index of community-acquired pneumonias, and is composed of the following dimensions: confusion (defined by a mental test score ≤ 8, new disorientation in person, place or time); blood urea nitrogen (BUN) > 20 mg/dL; respiratory rate ≥ 30 breaths/min, blood pressure (systolic < 90 mm Hg or diastolic ≤ 60 mm Hg), and age ≥ 65 years. Classification is as follows: 0–1: probably suitable for home treatment, low risk of death; 2: consider hospital-supervised treatment, and ≥ 3: manage at the hospital as severe pneumonia; high risk of death.

Sepsis-related organ failure assessment (4) is a system for assessing the onset and progression of multiorgan failure in intensive care unit (ICU) patients. It uses constants for six organs systems and for some treatment regimens (vasoactive agents): breathing (PaO_2_/FiO_2_), coagulation (platelets × 10^3^/mm^3^), liver (bilirubin), cardiovascular (hypotension), nervous system (Glasgow Coma Scale), renal (creatinine mg/dl). Each of the organs is assigned a score from 0 to 4. The score is the sum of all the individual organ assessments. A score different from 0 to < 3 is evaluated as organ dysfunction, while higher scores indicate organ failure. The total score was considered for the purposes of this study.

The acute physiology and chronic health evaluation II (4) is a prognostic classification system that stratifies patients using a score based on the baseline values of 12 physiological measurements [heart rate (HR), rectal temperature, arterial pH, Glasgow Coma Scale, oxygenation (FiO_2_), bicarbonate (HCO_3_), creatinine, leukocytes/mm^3^, serum Na, serum K, age, and previous health status] to provide an overall measure of disease severity. The higher the score (range 0–71) the greater the severity. For the purposes of this study the total score was considered.

CO-RADS scale: a categorical CT assessment scheme for patients with suspected COVID-19, this scale should be used in patients with moderate to severe symptoms. Set seven categories: (1) CO-RADS 0 (not interpretable, the technique was insufficient to assigning a score), (2) CO-RADS 1 (very low, normal, or non-infectious), CO-RADS 2 (low, typical findings for another infection, but not COVID-19), (3) CO-RADS 3 (equivocal/unsure, characteristics compatible with COVID-19 but also with other diseases), (4) CO-RADS 4 (high suspicion of COVID-19), (5) CO-RADS 5 (very high, typical for COVID-19), and (6) CO-RADS 6 [proven, real time polymerase chain reaction (RT-PCR) for SARS-CoV-2] (15).

Other health conditions: comorbidity was determined using the Charlson comorbidity index and the total score was divided in to groups: high comorbidity ≥ 3 and low comorbidity ≤ 2 points (16, 17). Sociodemographic variables included sex, age in years, years of education, employment status, and occupation.

### Statistical analysis

In the descriptive analysis, means ± SD were used for continuous variables, as well as frequencies and percentages for categorical variables. For the comparison of variables between groups (those who died and those who did not), *t*-tests were used for continuous variables and Chi^2^ for categorical variables. A multivariate analysis was performed including raw values adjusted with Cox regression to see the association of PA and MS with mortality adjusting for other variables. The graphic representation of survival was performed using Kaplan–Meier curves. These analyses were carried out using the STATA software, version 16. Sample size was calculated considering our hypothesis that PA will be associated with mortality. We use the findings of Cornejo-Pareja et al. (7) they reported an OR of 2.48 between PA and mortality, with a mortality rate of 32.4% in the low PA group versus the normal PA group (6.5%). Considering an alpha error of 0.05, a power of 80% and a loss rate of 10%, requiring a minimum of 78 patients, we recruited *n* = 104 patient.

### Ethics statements

This study was reviewed and approved by the Bioethics Committee of General Hospital Penjamo, Guanajuato, Mexico. The patients/participants provided their written informed consent to participate in this study.

## Results

The mean age of the patients was 62.7 ± 15.0 (31–96), 48.1% were females (*n* = 51). A total of 59.6% had less than 10 years of education and 36.5% did not have a job. The most common pre-existing diseases were diabetes without complications (32.08%), chronic obstructive pulmonary disease (COPD) (10.3%), diabetes with complications (6.6%), and kidney failure (2.8%). As regards the baseline body composition and anthropometric data, the following averages were obtained: FMI was 12.71 ± 5.5 kg/m^2^, AMMI was 7.4 ± 1.5 kg/m^2^, PA was 5.1° ± 1.6°, weight was 81.3 ± 18.4 kg, and BMI was 30.2 ± 6.7 kg/m^2^. The mean of grip strength was 14.1 ± 10.7 kg. At the time of admission, the scores in the prognostic scales used-SOFA, APACHE II, and CURB-65-were, respectively, as follows: 4 ± 1.6, 8.9 ± 4.7, and 2.3 ± 1.03 (Table 1).

**TABLE 1:** Baseline characteristics: sociodemographic, clinical, anthropometry, and body composition and disease severity scales.

Baseline characteristics	Total *n* = 104 Mean ± SD or *n*, %	Participants who died *n* = 42 (40.4%) Mean ± SD or *n*, %	Participants who survived *n* = 62 (59.6%) Mean ± SD or *n*, %	*p*-value
Age (years)	62.79 ± 15.02	66.81 ± 14.78	59.9 ± 14.83	0.0216
**Gender**				
Females	51 (48.11%)	16 (38.10)	34 (54, 84)	0.094
**Education**				
1–10 years	62 (59.62%)	24 (57.14)	38 (61, 29)	
> 10 years	9 (8.65%)	3 (7.14)	6 (9.68)	0.737
**Occupation**				
Not employed	38 (36.54%)	19 (45.24)	19 (30.65)	0.129
Charlson index ≥ 3	8 (7.55)	5 (59.62)	3 (37.50)	0.030
**Anthropometry and body composition**				
BMI, kg/m^2^	30.23 ± 6.73	30.60 ± 6.35	29.98 ± 7.02	0.6513
Fat mass index kg/m^2^	12.71 ± 5.59	11.80 ± 6.32	13.34 ± 5.02	0.2829
Appendicular muscle mass index kg/m^2^	7.44 ± 1.51	6.03 ± 1.29	7.25 ± 0.92	0.0148
Phase angle°	5.10 ± 1.60	4.81 ± 1.72	5.43 ± 1.53	0.0309
Phase angle ≤ 3.66°	27 (25.96)	17 (40.48)	10 (16.13)	0.0055
Grip strength, kg/force	14.18 ± 10.72	9.33 ± 9.82	16.61 ± 10.39	0.0000
Grip strength, ≤ 5.0 kg/force	55 (56.12)	21 (51.22)	34 (59.65)	0.4068
**Disease severity instruments**				
CO-RADS (total score)	4.61 ± 0.71	4.83 ± 0.65	4.46 ± 0.09	0.0098
SOFA (total score)	4 ± 1.64	4.64 ± 2.10	3.56 ± 1.06	0.0008
APACHE II (total score)	8.96 ± 4.76	11.66 ± 5.18	7.10 ± 3.42	0.0000
CURB-65 (total score)	2.38 ± 1.03	2.78 ± 1.09	2.11 ± 0.94	0.0009

APACHE II, the acute physiology and chronic health evaluation II; SOFA, sepsis-related organ failure assessment; CURB-65, score for pneumonia severity; CO-RAD, a categorical CT assessment scheme for patients with suspected COVID-19; BMI, body mass index; AMMI, appendicular muscle mass index; PA, phase angle, MS, muscle strength.

In the comparison between the group of patients who died and those who did not, the following variables were significant: age was higher in those who died, 66.8 ± 14.7, vs. those who survived, 59.9 ± 14.3, *p* = 0.0216. The group that died also had a higher comorbidity index (Charlson index ≥ 3 in the total score) of 59.6% vs. the group that survived, whose index was 37.5%, *p* = 0.030. The significant body composition variables were: AMMI, which showed that the group that died had lower values, 6.03 ± 1.2, vs. 7.2 ± 0.9 in the group that survived, *p* = 0.0148. PA values were also lower in the group that died, 4.8 ± 1.7, vs. 5.43 ± 1.5 in the group that survived, *p* = 0.0309. MS was also lower in the group that died vs. the group that survived (9.3 ± 9.8 vs. 16.6 ± 10.3, *p* = 0.0010). In the prognostic scales, the scores were higher in the group that died: SOFA (4.6 ± 2.1 vs. 3.56 ± 1.06, *p* = 0.0008); APACHE II (11.6 ± 5.1 vs. 7.1 ± 3.4, *p* = 0.0000) and CURB-65 (2.7 ± 1.09 vs. 2.1 ± 0.94, *p* = 0.0009) (Table 1). CO-RADS scale (4.8 ± 0.6 vs. 4.4 ± 0.09, *p* = 0.009).

Table 2 shows the results of the Cox regression model, which indicate that the variables that were significantly associated with mortality were: a low PA (HR = 2.2, 95% CI = 1.2–5.1), a low MS (HR = 3.6, 95% CI = 1.6–8.0), and the APACHE II score (HR = 1.1, 95% CI = 1.0–1.2). All of them remained associated with mortality, regardless of sex, age, comorbidity, other prognostic scales (CURB-65 and SOFA) and AMMI (Table 2).

**TABLE 2:** Association between low phase angle, low muscle strength, and severity scales with mortality.

Variables	Unadjusted HR	95% CI	*p*	Adjusted HR	95% CI	*p*
Sex (male)	1.504	0.799–2.833	0.206	1.458	0.6637–3.204	0.348
Age (years)	1.026	1.002–1.051	0.029	1.005	0.9722–1.039	0.761
APACHE II (total score)	1.113	1.054–1.174	0.000	1.162	1.063–1.270	0.001
CURB-65 (total score)	1.433	1.036–1.981	0.029	0.943	0.593–1.498	0.805
SOFA (total score)	1.125	0.982–1.287	0.087	0.876	0.6768–1.134	0.317
CO-RADS (total score)	1.705	1.004–2.896	0.048	1.969	1.095–3.541	0.024
Charlson index (score ≥ 3)	1.659	0.506–5.433	0.403	0.690	0.191–2.488	0.573
Low phase angle ≤ 3.66°	2.957	1.517–5.765	0.001	2.571	1.217–5.430	0.013
Appendicular muscular mass index (kg)	0.950	0.781–1.15	0.611	0.9292	0.728–1.1847	0.554
Muscle strength ≤ 5.0 kg/force	3.390	1.742–6.599	0.000	4.519	1.992–10.252	0.000

APACHE II, the acute physiology and chronic health evaluation II; SOFA, sepsis-related organ failure assessment; CURB-65, score for pneumonia severity; CO-RAD, a categorical CT assessment scheme for patients with suspected COVID-19; BMI, body mass index; AMMI, appendicular muscle mass index; PA, phase angle, MS, muscle strength.

**FIGURE 1:**
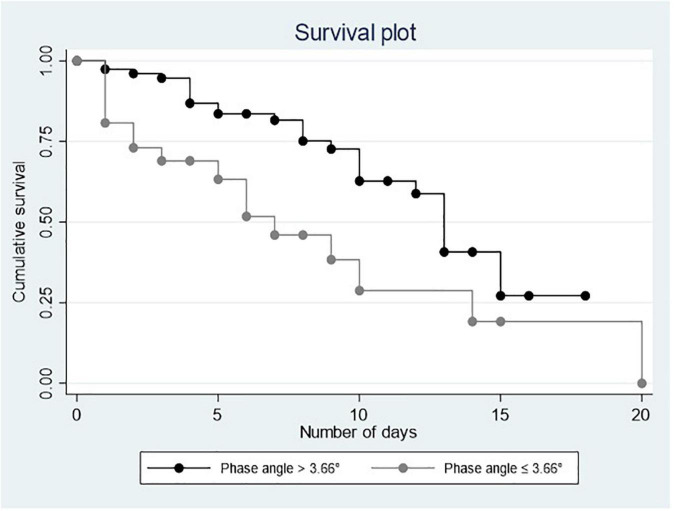
Survival plots for phase angle.

**FIGURE 2:**
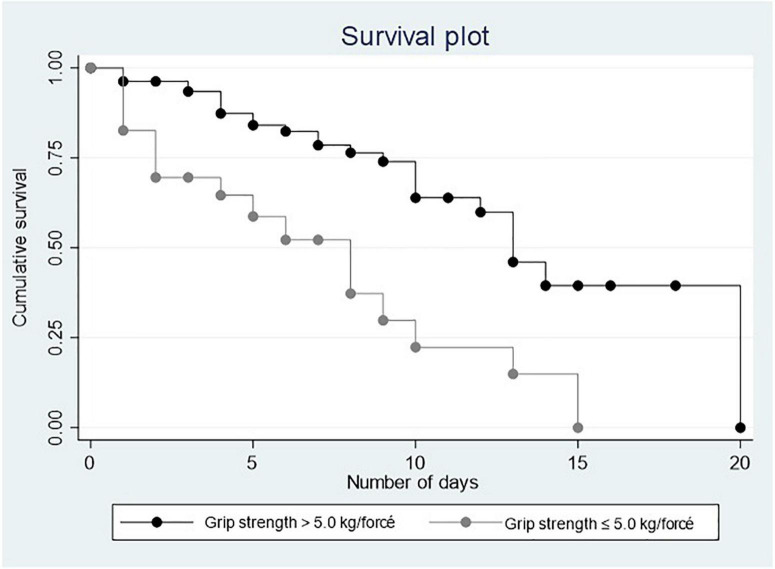
Survival plots for muscle strength.

Figures 1, 2 show the Kaplan–Meier curves that depict a positive relationship of PA and MS with survival.

## Discussion

The main results of this study demonstrate that a low PA (≤ 3.66°), HR = 2.5, (95% CI: 1.2–5.4), and that a low MS (≤ 5.0 kg/force), HR = 4.5, (95% CI: 1.9–10.2) represents a higher risk of mortality in hospitalized SARS-CoV-2 patients than the prognostic and severity scales prognostics; APACHE, HR = 1.1 (95% CI: 1.0–1.2), CURB-65 and SOFA were not significant. To our knowledge, no study has compared the three main predictive scales for mortality in critically ill patients (APACHE; CURB-65 and SOFA) against low MS, low PA, and other body composition assessments (FMI, AMMI, and BMI). Several reasons could be explain the lower risk shown by the prognostic scales in this study; all the evaluations were made at the admission of the patients, scales such as APACHE, SOFA, and CURB-65 include physical and biochemical parameters that can change constantly. As an example, SOFA which was designed and includes parameters of organ failure (liver, kidney, central nervous system, lung), which could occur in a severe phase of the COVID-19 disease (few days before death) (18).

The acute physiology and chronic health evaluation is a very complete scale that includes different parameters, physical, vital signs, biochemical, and complete blood count; however, hematocrit or a blood low pH are not good predictors of mortality in COVID-19 early stage or at admission. Moreover, leukocytosis is related to bacterial pneumonia (6). About the age, it is included into CURB-65 and APACHE, in our sample we included people aged 40 years and older, which might affect the risk predictive to mortality. Further studies including trajectories analysis are needed to confirm the mechanisms underlying these findings (18).

About the association between a low PA and mortality are consistent with previous studies (8, 9). Our results agree with previous studies that low MS is a good predictor of mortality (13, 18). Therefore, low MS plays a key role in recovery from critical illness, especially from SARS-CoV-2 infection, as strength, which is an indicator of muscle function, is key during the disease process and recovery (19). In case of pre-existing or early MS impairment prior to the onset of acute illness, hospitalization may make it worse and lead to the patient not recovering normal muscle function and being at increased the risk of developing cachexia during the inflammatory process and sarcopenia at a later stage (once the acute inflammation subsides) (18, 19). Our study not found association with low muscle mass measured by AMMI, similar to the findings of Osuna-Padilla et al. (9) who did not find an association with mortality. The difference between muscle quantity and quality has been attributed to the fact that the low MS is a combination of neural and muscular factors, such as deficiencies in neural activation, the need for voluntary capability to activate the required muscular system, and the fact that low MS may occur earlier than of mass muscle loss, which makes it more sensitive to change and may explain its association with mortality in patients with COVID-19 pneumonia (20–22).

Regarding the low PA, it is a mixed indicator composed of resistance, reactance and capacitance, these parameters are directly related to the conduction of the electrical current through the intercellular, interstitial and intracellular spaces; the great inflammatory state that characterizes COVID-19 pneumonia promotes a greater resistance to the passage of the electrical current through different tissues, including muscle tissue in the early stages of the disease, this could explain why the PA is remained associated as a risk predictor for mortality in this study and similar studies (7, 8).

The association between the FMI and the BMI with mortality was not demonstrated. This lack of association is consistent with the results in several studies included in an interesting systematic review, (23) in which it is shown that there is an association between the high fat mass with hospital admission, severity of the disease, orotracheal intubation, among others, but with a contradictory association with mortality.

The limitations of this study should be borne in mind. The first one is that mortality was the only negative outcome studied; future studies derived from this cohort or other cohorts should consider whether a low PA and a low MS have a higher risk to predict a longer hospital stay and need for intubation versus APACHE, CURB-65, and SOFA. Another limitation is that the cut-off points for PA and low MS were similar to others studies above mentioned but are only representative of people with the following characteristics: Mexican adults hospitalized with the diagnosis of SARS-CoV-2 pneumonia, adults aged from 31 to 96 years, males and females. However, the main strength of this study is one of the first to demonstrate that a low PA and a low MS upon admission are higher risk predictors of mortality than prognostic scales such as SOFA, APACHE II, and CURB-65. Further studies with trajectories analysis for MS and PA, are needed to demonstrate whether changes in body composition remain as predictors beyond hospital admission.

## Conclusion

Study results suggest that low MS and low PA are greater risk predictors for mortality than APACHE, SOFA, and CURB-65 indexes, at hospital admission. It is recommended to include them as part of the initial evaluation in all patients admitted with pneumonia due to COVID-19, it will help to implement interventions aimed at improving strength and PA to reduce the risk of mortality.

## Data availability statement

The raw data supporting the conclusions of this article will be made available by the authors, without undue reservation.

## Ethics statement

The studies involving human participants were reviewed and approved by the Bioethics Committee of General Hospital Penjamo, Guanajuato, Mexico. The patients/participants provided their written informed consent to participate in this study.

## Author contributions

OR-C: conceptualization and supervision. OR-C and ML-T: methodology. ML-T and GN-F: formal analysis. PA-M, OR-C, JC-O, AN-C, and GD-M: investigation. ML-T, OR-C, GN-F, and PA-M: writing—original draft. All authors contributed to the writing, review, and editing.
